# Impaired Spinal Glucocorticoid Receptor Signaling Contributes to the Attenuating Effect of Depression on Mechanical Allodynia and Thermal Hyperalgesia in Rats with Neuropathic Pain

**DOI:** 10.3389/fncel.2017.00145

**Published:** 2017-05-19

**Authors:** Xiao Wei, Yuqi Sun, Fei Luo

**Affiliations:** ^1^Key Laboratory of Mental Health, Institute of Psychology, Chinese Academy of SciencesBeijing, China; ^2^Department of Psychology, University of Chinese Academy of SciencesBeijing, China

**Keywords:** glucocorticoid receptor, depression, neuropathic pain, dexamethasone, spinal dorsal horn

## Abstract

Although depression-induced altered pain perception has been described in several laboratory and clinical studies, its neurobiological mechanism in the central nervous system (CNS), particularly in the spinal dorsal horn, remains unclear. Therefore, in this study, we aimed to clarify whether nociceptive sensitivity of neuropathic pain is altered in the olfactory bulbectomy (OB) model of depression and whether glucocorticoid receptor (GR), which is involved in the etio-pathologic mechanisms of both major depression and neuropathic pain, contributes to these processes in the spinal dorsal horn of male Sprague-Dawley rats. The results showed that mechanical allodynia and thermal hyperalgesia induced by spinal nerve ligation (SNL) were attenuated in OB-SNL rats with decreased spinal GR expression and nuclear translocation, whereas non-olfactory bulbectomy (NOB)-SNL rats showed increased spinal GR nuclear translocation. In addition, decreased GR nuclear translocation with normal mechanical nociception and hypoalgesia of thermal nociception were observed in OB-Sham rats. Intrathecal injection (i.t.) of GR agonist dexamethasone (Dex; 4 μg/rat/day for 1 week) eliminated the attenuating effect of depression on nociceptive hypersensitivity in OB-SNL rats and aggravated neuropathic pain in NOB-SNL rats, which was associated with the up-regulation of brain-derived neurotrophic factor (BDNF), TrkB and NR2B expression in the spinal dorsal horn. The present study shows that depression attenuates the mechanical allodynia and thermal hyperalgesia of neuropathic pain and suggests that altered spinal GR-BDNF-TrkB signaling may be one of the reasons for depression-induced hypoalgesia.

## Introduction

Major depression and chronic pain are difficult to completely heal, have high comorbidity (Bair et al., 2003; Doan et al., 2015) and show clinical interactions with each other. Chronic pain patients are at higher risk of depression than healthy population (Ratcliffe et al., 2008). In addition, more than half of depression patients suffer from chronic pain (Bair et al., 2003). The impact of depression on chronic pain perception is a controversial topic. Some researchers have reported significant aggravation of pain stimuli aversion (Bravo et al., 2012) and increased spontaneous formalin pain (Shi et al., 2010b; Su et al., 2010) in rodent depressive-like models as well as deep somatic pain in depression patients (Bär et al., 2005; Su et al., 2010). Nonetheless, other clinical and laboratory studies found that depression patients showed reduced sensitivity to noxious stimuli applied to the skin (Lautenbacher et al., 1994; Bär et al., 2006). Depressive-like rats combining with chronic pain exhibited hypoalgesia to nociceptive mechanical and thermal stimuli compared to chronic pain rats without depression (Shi et al., 2010a,b; Wang et al., 2010). Studies on the mechanism of hypoalgesia induced by depression are currently focused on the physiopsychology of pain perception and the neural activity of supraspinal nuclei, such as the reduction of pain-avoidance motivation (Wang et al., 2016) and altered neural activity in the thalamo-cortical circuits of the medial/lateral pain pathway (Wang N. et al., 2013). However, whether nociceptive transmission and related neuropathological mechanisms are altered in the spinal dorsal horn under this circumstance is still unclear.

Major depression and chronic pain have several etio-pathologic mechanisms in common (Han and Pae, 2015). Glucocorticoid receptors (GRs), which belong to the classical nuclear receptor family, are widely distributed in the central nervous system (CNS; Fuxe et al., 1987; Shaqura et al., 2016). In the absence of glucocorticoids, GRs reside in the cell cytoplasm as part of a chaperone protein complex. Upon glucocorticoid binding, GRs dissociate from the chaperone complex, translocate into the nucleus through nuclear pores and regulate the expression of target genes leading to transcriptional activation/inhibition (Kadmiel and Cidlowski, 2013; Oakley and Cidlowski, 2013). In studies of the etiology and pathology of major depression, disorders of GR expression and function in the prefrontal cortex and several limbic brain areas (e.g., hippocampus and amygdala) were thought to be involved in hypothalamic pituitary adrenal axis (HPA axis) dysfunction, which is one of the potential mechanisms associated with the pathogenesis of depression (Pariante, 2006; Anacker et al., 2011a; Guidotti et al., 2013). In studies of the neural mechanisms of chronic pathological pain, researchers have found that enhanced spinal GRs contributed to central sensitization and allodynia/hyperalgesia of neuropathic pain induced by peripheral nerve injury, and GR antagonists could block the mechanical allodynia and/or thermal hyperalgesia of chronic inflammatory pain and neuropathic pain (Takasaki et al., 2005; Wang et al., 2005; Alexander et al., 2009; Le Coz et al., 2014; Zhang et al., 2014; Madalena and Lerch, 2016). Based on these findings, we speculated that spinal GR expression and function may participate in the neural mechanisms of depression-induced attenuation of chronic pain, especially neuropathic pain.

Brain-derived neurotrophic factor (BDNF) is an important neuropeptide that contributes to neural plasticity and neurogenesis in the CNS. Chronic stress-induced depressive-like behavior is associated with a reduction of GRs, BDNF expression and neuronal apoptosis in the hippocampus, which can be reversed by antidepressants (Numakawa et al., 2013; Xing et al., 2015). Local application of BDNF on the spinal dorsal horn induced long-time potentiation (LTP) of C-fiber evoked field potentials (Zhou et al., 2008) and the activation of p-SFKs and p-p38 MAPK signaling in microglia (Zhou et al., 2011) as well as TrkB signaling in neurons (Beggs and Salter, 2013; Khan and Smith, 2015), which is necessary for the central sensitization of neuropathic pain. However, to date, there has been little evidence of interactions between GR and BDNF signaling in the spinal dorsal horn. Therefore, we wondered whether spinal GRs participate in the influence of depression on neuropathic pain by regulating BDNF expression and related signaling pathways.

Bilateral olfactory bulbectomy (OB), a well-established animal model of depression (Redmond et al., 1997; Song and Leonard, 2005), results in a series of behavioral and neurochemical alterations comparable with those observed in depression patients, such as enhanced locomotor response to stress (e.g., enhanced locomotor and rearing behaviors in open field test; Redmond et al., 1997; Dandekar et al., 2009; Morales-Medina et al., 2012), decreased sucrose preference (Romeas et al., 2009; Stepanichev et al., 2016), extended floating time in the forced swim test (Morales-Medina et al., 2012; Zhang et al., 2016) and abnormal changes of brain serotonergic, noradrenergic, glutamatergic, dopaminergic and GABAergic systems (Song and Leonard, 2005). Although the mechanism of OB-induced depressive-like behavior is complex, most of the above changes can be reversed by chronic antidepressants treatment (Cryan and Mombereau, 2004; Song and Leonard, 2005), and it is one of the most reliable depression animal models, at least in rats (Willner and Mitchell, 2002). Therefore, in the present study, we used OB to establish depressive-like behavior in rats and then used spinal nerve ligation (SNL) to induce neuropathic pain. Mechanical allodynia and thermal hyperalgesia were investigated with von Frey fibers and radiant heat stimuli, respectively, to detect the effect of depression on neuropathic pain nociception. Then, immunofluorescence staining and Western blotting were implemented to investigate the expression and translocation of spinal GRs, and serum concentration of corticosterone was measured to assess the activity of HPA axis. Subsequently, the GR agonist dexamethasone (Dex) was administered intrathecally to confirm whether depression-induced nociceptive alternations were mediated by spinal GRs and whether BDNF signaling was modulated by GRs in this situation.

## Materials and Methods

### Experimental Protocol

**Figure 1:**
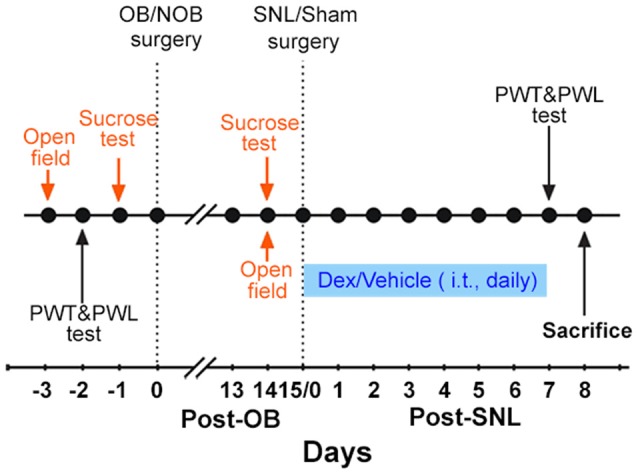
**Experimental protocol.** The protocol indicated in orange was only carried out in Experiment 1. The protocol indicated in blue was only carried out in Experiment 2. OB, olfactory bulbectomy; NOB, non-olfactory bulbectomy; SNL, spinal nerve ligation; PWT, paw withdrawal threshold; PWL, paw withdrawal latency; Dex, dexamethasone; i.t., intrathecal injection.

The present study consisted of two experiments. The main protocol is shown in Figure 1.

Experiment 1: first, the baselines of open field test, sucrose consumption test, paw withdrawal threshold (PWT) and paw withdrawal latency (PWL) were measured. Then, OB/Non-olfactory bulbectomy (NOB) surgery was performed. 14 days later, open field and sucrose tests were employed to assess depression-like behaviors. Subsequently, SNL/sham surgery was conducted on OB/NOB rats. Consequently, there were four groups of rats: NOB-Sham group, OB-Sham group, NOB-SNL group and OB-SNL group. Seven days post-SNL or sham surgery, PWT and PWL tests were conducted to assess the nociceptive behavior of rats. On the second day of the behavior tests, rats were sacrificed to obtain spinal tissues and blood samples. Immunofluorescence staining and Western blotting were performed to analyze protein expressions. Plasma corticosterone levels were measured by enzyme linked immunosorbent assay (ELISA).

Experiment 2: rats were treated as in Experiment 1 before the SNL/sham operation. Starting on the SNL/sham surgery day, intrathecal injection (i.t.) of Dex/vehicle was performed daily for1 week. PWT and PWL tests were conducted to assess nociceptive behavior within 30 min following the last drug delivery. On the second day of behavior tests, spinal tissues were obtained to perform Western blotting.

### Animals

Male Sprague-Dawley rats (220–290 g) from the Laboratory Animal Center of the Academy of Military Medical Science (Beijing, China) were used. Rats were housed in separate cages, and the room was kept at 22 ± 2°C under a 12/12 h light/dark cycle (lights on at 7:00 am) and with free access to food and water. Rats were acclimatized for 5–7 days before experiments and handled 2–3 min per day before surgeries and behavior tests. This study was carried out in accordance with the guidelines of the National Institutes of Health on experimental animal care and use. All experimental procedures were approved by the Institutional Review Board of the Institute of Psychology, Chinese Academy of Sciences.

### Olfactory Bulbectomy Surgery

Bilateral OB was employed to induce depressive-like behaviors. Animals were anesthetized with 1% sodium pentobarbital (50 mg/kg, i.p.) and then fixed onto a brain stereotactic apparatus. According to Kelly and Leonard’s method (Kelly et al., 1997), a midline sagittal cut was made on the head skin, 30% H_2_O_2_ was used to clean the tissue, and the skull was exposed. Two boreholes were made 8 mm rostral to the bregma, 2 mm lateral to the midline separately. Bilateral olfactory bulbs were removed using a vacuum suction pump through two boreholes. The cavity was quickly filled with gelatin sponge. Penicillin (160,000 U) was injected intraperitoneally after surgery. Rats in NOB group received the same operation without removing any brain tissue. At the end of all the experiments, animals were dissected to confirm whether the bilateral OB was successful. If the olfactory bulbs were removed incompletely or the prefrontal cortex was damaged, the relevant data were excluded.

### Intrathecal Injection and Spinal Nerve Ligation Surgery

For intrathecal injection, rats were anesthetized, and the skin above the posterior superior iliac spine was shaved and incised. According to Storkson’s method (Størkson et al., 1996), a sterile polyethylene-10 (PE-10) catheter filled with sterile saline was inserted through the L5/L6 intervertebral space until the tip of the catheter reached the spinal lumbar enlargement level. The PE-10 catheter was fixed and led out from the neck skin through a subcutaneous tunnel.

Then, SNL was applied to establish neuropathic pain according to Kim and Chung’s method (Kim and Chung, 1992). The muscles were dissected until the left spinal L5 transverse was exposed and removed. The L5 spinal nerve was isolated and ligated with silk thread distal to the L5 DRG. Animals in sham group underwent the same operation except the ligation of L5 spinal nerve. The wound was sutured in two layers and disinfected with iodophor and 75% ethyl alcohol.

Drug or vehicle was delivered through the intrathecal catheter. Dex (Sigma, St. Louis, MO, USA) was dissolved with ethyl alcohol to a 5 mg/ml stock solution. Before administration, stock solution was diluted with saline to a 0.5 mg/ml working solution. Each animal received 8 μl (4 μg) working solution of Dex or vehicle (10% ethanol in saline) and then 12 μl saline to ensure that drug was completely delivered into the subarachnoid space. The first administration of the drug or vehicle was performed 30 min before SNL surgery. Subsequently, the drug or vehicle was administered once daily for 7 days. After all the behavior tests were completed, successful catheterization was confirmed immediately by bilateral hind limb paralysis following injection of 20 mg/ml lidocaine (8 μl) through the catheter within 30 s.

### Behavior Tests

Open field test was carried out to analyze rats’ horizontal movements and exploratory behaviors before and after OB surgery. The open field device was composed of a circular area (180 cm in diameter with a 50 cm-high wall). In a quiet environment with dim illumination (40 w), each rat was tested in the open field for 5 min. The movement distance during the test was recorded and analyzed with a computer-based system Etho Vision (Noldus Information Technology, Wageningen, Netherlands). The number of rearing behavior was counted by the experimenter. The device was cleaned with 75% ethanol to remove olfactory cues in the interval between tests.

Sucrose consumption test was used to assess the rats’ anhedonia before and after OB surgery. In the preparation period, rats were given a bottle of water and a bottle of 1% sucrose solution simultaneously for the first 24 h (with food). Then, the two bottles were switched, and another 24 h were given to distinguish the sucrose solution and water (with food). Immediately thereafter, the rats were subjected to 22 h of food and water deprivation. Subsequently, formal sucrose preference test was performed: each rat was presented simultaneously with 1% sucrose solution bottle and water bottle for 1 h (with food). Each rat’s consumption of 1% sucrose solution and water were recorded. The percent preference for sucrose consumption was calculated using the following formula:

Sucrose preference = (sucrose solution consumption/[sucrose solution consumption + water consumption]) × 100%.

PWTs in response to mechanical stimuli were measured with electronic von Frey fiber to assess rats’ mechanical allodynia. Rats were placed on a metal grid, and the plantar skin of the ipsilateral hind paw of SNL/sham surgery was stimulated with electronic von Frey fiber with a 5 min interval. The mechanical withdrawal threshold was recorded when the rat was quickly withdrawing the hind paw. Three measurements of PWT were averaged as the result per test.

Thermal hypersensitivity was tested using radiant heat. Rats were placed on a glass floor and separated by Plexiglas covers. A radiant heat source beneath the floor was aimed at the plantar surface of the ipsilateral hind paw of SNL/sham surgery. The PWL induced by heat was used as a measure of thermal hyperalgesia. Three measurements of latency with 5 min interval were conducted for each rat in each test session. The average of three measurements was the final result of each rat per test.

### Serum Corticosterone Concentration

To minimize the circadian effects on corticosterone concentration, at the second day of the final PWT and PWL tests in Experiment 1, orbital blood samples of rats were collected for corticosterone determination from 8:00 to 11:00 in the morning. The blood were centrifuged at 3000 rpm for 10 min to obtain serum and preserved at –80°C. The serum concentrations of corticosterone were measured by ELISA using a commercial ELISA kit (R&D Systems Inc., Minneapolis, MN, USA).

### Immunofluorescence Staining

After being anesthetized with 10% chloral hydrate (400 mg/kg, i.p.), the rats were perfused with cold saline and then 4% paraformaldehyde in 0.1 M phosphate buffer (pH 7.2–7.4) through the ascending aorta and the lumbar enlargement of the spinal cord was removed and post fixed in 4% paraformaldehyde for 1 h and then transferred into 30% sucrose in 0.1 M phosphate buffer for 3 days. Transverse spinal sections (20 μm) were cut on a cryostat (Leica CM3035 S; −20°C to −22°C). Subsequently, free-floating sections were blocked with 3% donkey serum in 0.3% Triton X-100 for 1 h at room temperature and incubated with different primary antibodies: polyclonal GR (1:200, Santa Cruz Biotechnology, sc-1004, Santa Cruz, CA, USA), monoclonal NeuN (neuronal marker, 1:200, Abcam, ab104224, Cambridge, UK), monoclonal GFAP (astrocyte marker, 1:1000, Cell Signaling Technology, #3670, Beverly, MA, USA) or polyclonal Iba1 (microglia marker, 1:500, Abcam, ab5076, Cambridge, UK) overnight at 4°C. Sections were washed with 0.01 M PBS for 10 min three times and then incubated with FITC (1:200; Jackson Immunolab, 115-095-003, 705-095-003, West Grove, PA, USA) and TRITC-conjugated secondary antibodies (1:200; Jackson Immunolab, 711-025-152, West Grove, PA, USA) for 1 h at room temperature.

Stained sections were observed with a Leica DM5500B (Leica Camera AG, Solms, Germany) fluorescence microscope, and images were captured with a CCD spot camera. Double-stained images were merged using Image J software. Semi-quantitative analysis of GR-positive areas and areas of colocalization between GRs and cell markers in the ipsilateral spinal dorsal horn were carried out with Image-Pro Plus 5.0 (Media Cybernetics, Rockville, MD, USA). The colocalization rate of GRs and neurons/astrocytes/microglia was calculated according to the following formula:

Colocalization rate = (positive area of both GR and cell markers/positive area of cell markers) × 100%.

### Western Blotting

Spinal lumbar enlargement was harvested from anesthetized animals. The tissues were placed in liquid nitrogen immediately, and the dorsal part of the ipsilateral side was separated immediately and preserved at −80°C. For whole-cell protein extraction, tissues were homogenized in SDS lysis buffer (Beyotime, Shanghai, China) with PMSF (Thermo Scientific, Waltham, MA, USA), then sonicated on ice and centrifuged at 13,000× *g* for 15 min at 4°C to isolate the supernatant part. For cytoplasm and nuclei protein extraction, tissues were treated with an NE-PER Nuclear and Cytoplasmic Extraction Reagents Kit (Pierce Biotechnology, Rockford, IL, USA). The protein concentrations of all samples were detected with a BCA Protein Assay kit (Pierce Biotechnology, Rockford, IL, USA).

Proteins were separated by gelelectrophoresis (SDS–PAGE) and transferred onto a PVDF membrane (Bio-Rad). The membrane was blocked with 5% w/v nonfat dry milk or bovine serum albumin (BSA) in TBST (20 mM Tris-base, pH 7.6, 137 mM NaCl and 0.1% Tween 20) for 1 h at room temperature and then incubated with the primary antibodies polyclonal GR, polyclonal BDNF, polyclonal TrkB (1:200, Santa Cruz Biotechnology, sc-1004, sc-20981, sc-8316, Santa Cruz, CA, USA), polyclonal NR2B (1:1000, Cell Signaling Technology, # 4207, Beverly, MA, USA), monoclonal GAPDH, polyclonal histone H3 (1:200; Santa Cruz Biotechnology, sc-137179, sc-10809, Santa Cruz, CA, USA) overnight at 4°C with gentle shaking. The blots were washed for 10 min three times with washing buffer (TBST) and then incubated with secondary antibody horseradish peroxidase (HRP)-conjugated IgG (1:5000, # 7074, # 7076, Cell Signaling Technology, Beverly, MA, USA) for 1 h at room temperature. Subsequently, the membrane was washed for 10 min three times with TBST. Protein bands were detected by ECL (Pierce Biotechnology, Rockford, IL, USA) and exposed in a FluorChem™ E system (ProteinSimple, San Jones, CA, USA). Integrated optical densities were analyzed with the AlphaView software (Proteinsimple, San Jones, CA, USA).

### Statistics

All of the data are presented as mean ± SEM. Two-sample *t*-tests were employed to analyze data of the open field test, sucrose consumption test and rats’ bodyweights. Data of mechanical PWT and thermal PWL tests, serum corticosterone level, immunofluorescence staining and Western blotting affected by two or three factors were analyzed with a multifactor analysis of variance (ANOVA) with Bonferroni’s *post hoc* test. Correlations between mechanical PWT/thermal PWL and nuclear GR expression were examined with Pearson correlation coefficients. Statistical tests were performed with SPSS 19.0 (IBM, Armonk, NY, USA), and *p* < 0.05 was considered significant.

## Results

### Olfactory Bulbectomy Induced Abnormal Behaviors Correlated with Depressive-Like State and Attenuated Mechanical Allodynia and Thermal Hyperalgesia Caused by SNL

**Figure 2:**
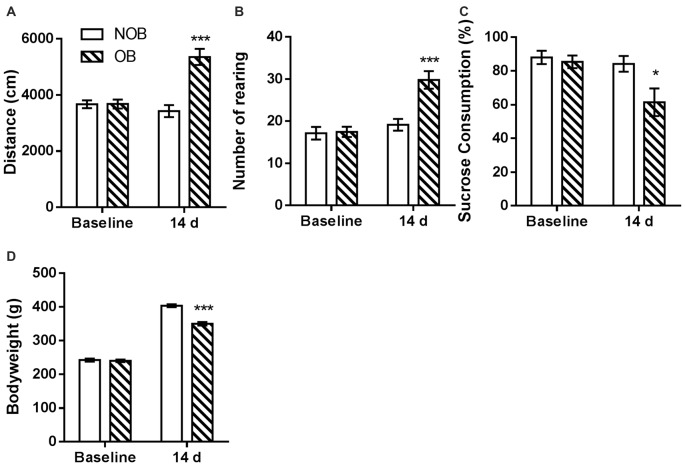
**OB induced depressive-like behaviors.** The OB group showed increased locomotor activity **(A)** and rearing numbers **(B)** in open field test, and decreased sucrose consumption **(C)** and bodyweight gain **(D)** compared to the NOB group at day 14. *p < 0.05, ***p < 0.001 vs. corresponding NOB group; *n* = 8–9 per group.

Behavioral alterations in the open field and sucrose tests and changes in the bodyweights of the rats were observed following a 14-day recovery phase after OB/NOB surgery. As shown in Figure 2, in open field test significantly more locomotor activity (Figure 2A) and rearing behavior (Figure 2B) were observed in OB rats than those in NOB rats (*p* < 0.001). As shown in Figure 2C, the sucrose solution consumption of OB rats was lower than that of NOB rats in the sucrose preference test (*p* = 0.034), revealing anhedonic symptom of depressive-like behavior. Furthermore, a significant reduction of bodyweight gain (Figure 2D) was observed in the OB group 14 days following OB surgery (*p* < 0.001). These results indicated that a depressive-like state was established following OB surgery.

**Figure 3:**
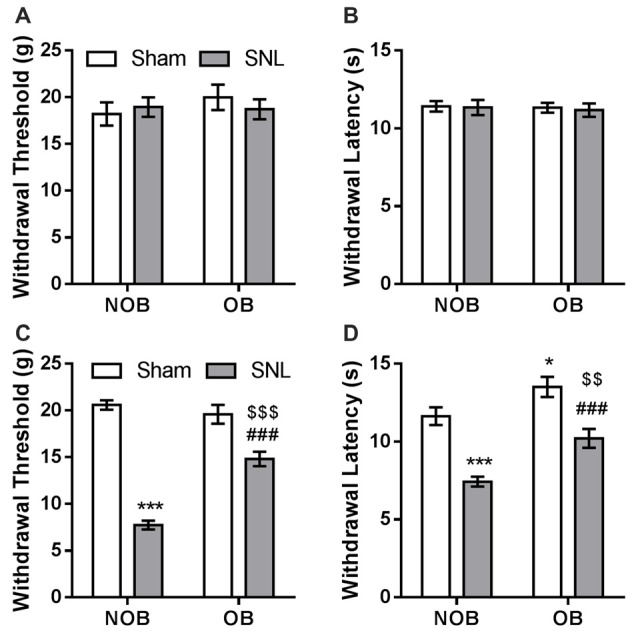
**SNL-induced mechanical allodynia and thermal hyperalgesia were attenuated by OB.** There was no differences found in the baselines of mechanical PWT **(A)** and thermal PWL **(B)** between four groups. Mechanical PWT **(C)** and thermal PWL **(D)** were decreased in the NOB-SNL group and partially restored in the OB-SNL group, which was lower than that in the OB-Sham group. *p < 0.05, ***p < 0.001 vs. NOB-Sham group; ^###^*p* < 0.001 vs. OB-Sham group; ^$$^*p* < 0.01, ^$$$^*p* < 0.001 vs. NOB-SNL group; *n* = 8 per group.

Subsequently, SNL/Sham surgery was performed on the OB/NOB rats. Seven days later, mechanical allodynia and thermal hyperalgesia were investigated using electronic von Frey fiber and thermal stimuli. There was no differences found in the baselines of mechanical PWT and thermal PWL between four groups (Figures 3A,B). As shown in Figure 3C, the mechanical PWT of the ipsilateral hind paw was significantly affected by OB and SNL (*F*_OB(1,28)_ = 17.635, *p* < 0.001, *F*_SNL(1,28)_ = 148.774, *p* < 0.001), and there was a significant OB × SNL interaction (*F*_OB × SNL(1,28)_ = 31.147, *p* < 0.001). NOB-SNL rats showed a lower PWT than NOB-Sham rats (*p* < 0.001), revealing mechanical allodynia associated with neuropathic pain. In the OB-SNL group, the PWT was also down-regulated by SNL (*p* < 0.001), but showed a restoration compared with NOB-SNL group (*p* < 0.001). In addition, there was no difference between the PWTs of the NOB-Sham group and the OB-Sham group (*p* = 0.337). As shown in Figure 3D, the thermal PWL of the ipsilateral hind paw was significantly regulated by OB and SNL (*F*_OB(1,28)_ = 17.654, *p* < 0.001, *F*_SNL(1,28)_ = 46.086, *p* < 0.001) without interaction (*F*_OB × SNL(1,28)_ = 0.658, *p* = 0.424). Similar to the results for the mechanical PWT, the thermal PWLs of both NOB-SNL group and OB-SNL groups were lower than those of their corresponding sham groups (*p* < 0.001), while the PWLs of both OB-Sham group and OB-SNL group were significantly higher than those of their corresponding NOB groups (*p* = 0.023, *p* = 0.001, respectively). These results revealed that the mechanical allodynia and thermal hyperalgesia of SNL rats were attenuated by an OB-induced depressive-like state.

### Decreased Spinal GR Expression and Translocation were Involved in the Depression-Induced Attenuation of Mechanical Allodynia and Thermal Hyperalgesia in Rats with Neuropathic Pain

#### The Expression and Translocation of GRs in the Spinal Dorsal Horn as Well as Serum Corticosterone Level Were Decreased and Associated with the Attenuation of Neuropathic Pain in Depressed Rats

**Figure 4:**
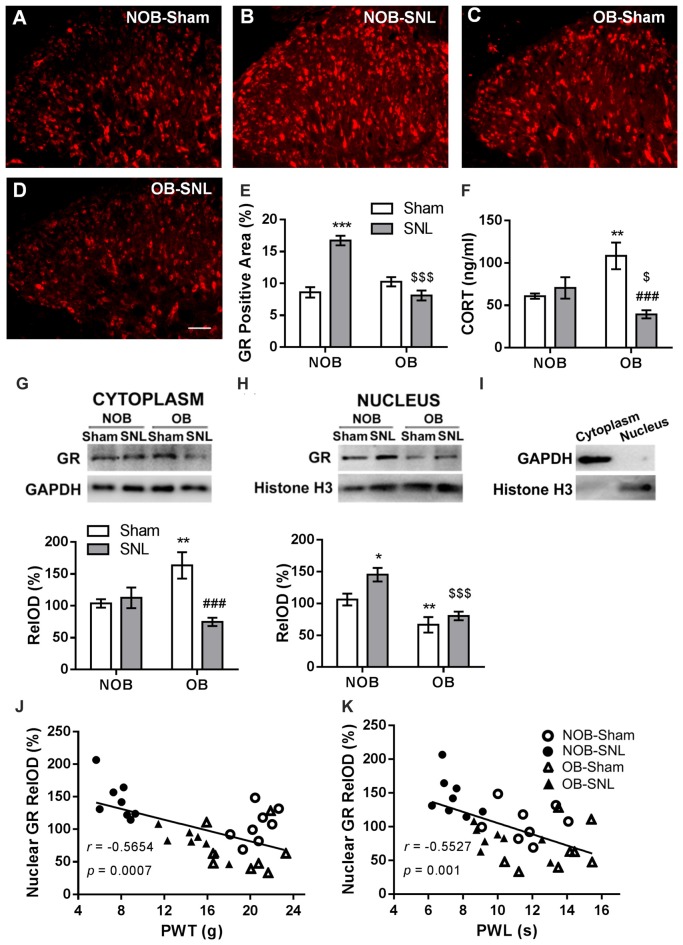
**Alterations of spinal glucocorticoid receptor (GR) expression, translocation and serum corticosterone level in NOB/OB-Sham/SNL rats.** The immunofluorescence-stained GR-positive area in the SNL-ipsilateral spinal dorsal horn increased in NOB-SNL rats but decreased in OB-SNL rats **(A–E)**; *n* = 6 sections of 3 rats per group; scale bar = 50 μm. Western blot results of spinal cytosolic and nuclear compartments showed enhanced cytosolic GR expression in the OB-Sham group and nuclear GR expression in the NOB-SNL group, while there was inhibited cytosolic and nuclear GR expression in the OB-SNL group **(G-I)**. *p < 0.05, **p < 0.01, ***p < 0.001 vs. NOB-Sham group; ^###^*p* < 0.001 vs. OB-Sham group; ^$$$^*p* < 0.001 vs. NOB-SNL group; *n* = 8 per group. Nuclear GR expression was negatively correlated with both mechanical PWTs **(J)** and thermal PWLs **(K)** of the NOB-Sham, NOB-SNL, OB-Sham and OB-SNL groups. Serum corticosterone concentration increased in the OB-Sham group but decreased in OB-SNL group **(F)** **p < 0.01 vs. NOB-Sham group; ^###^*p* < 0.001 vs. OB-Sham group; ^$^*p* < 0.05 vs. NOB-SNL group; *n* = 6 per group. CORT, corticosterone.

To determine the role of spinal GRs in the OB-induced attenuation of mechanical allodynia and thermal hyperalgesia of neuropathic pain, immunofluorescence staining and Western blotting were used to analyze GR expression and translocation in the present study. Semi-quantitative analysis of the immunofluorescence staining of GRs (Figures 4A–E) showed that there was a significant effect of OB and SNL on GR expression in the ipsilateral spinal dorsal horn (*F*_OB(1,20)_ = 20.336, *p* < 0.001; *F*_SNL(1,20)_ = 14.975, *p* = 0.001), with OB × SNL interaction (*F*_OB × SNL(1,20)_ = 44.456, *p* < 0.001). The spinal GR expression of NOB-SNL rats was prominently increased (*p* < 0.001), while GRs in the OB-Sham group were not changed compared with those in the NOB-Sham group (*p* = 0.143). Importantly, spinal GR level of the OB-SNL group was lower than that in the NOB-SNL group (*p* < 0.001).

Next, we analyzed GR protein levels in the cytosolic (Figure 4G) and nuclear (Figure 4H) compartments of the spinal dorsal horn by Western blotting to determine whether GR translocation was altered in the process. As shown in Figure 4G, in the cytosolic compartment of the spinal dorsal horn, there was a significant effect of SNL (*F*_OB(1,28)_ = 0.617, *p* = 0.439; *F*_SNL(1,28)_ = 8.233, *p* = 0.008) with OB × SNL interaction (*F*_OB × SNL(1,28)_ = 12.269, *p* = 0.002) on the GR level. OB resulted in a higher cytosolic GR level in OB-Sham group than in NOB-Sham group (*p* = 0.005), revealing an accumulation of spinal cytosolic GRs in depressed rats. Moreover, the cytosolic GR level was lower in the OB-SNL group than that in the OB-Sham group (*p* < 0.001). As shown in Figure 4H, GR protein level in the nuclear compartment was significantly regulated by OB and SNL (*F*_OB(1,28)_ = 27.669, *p* < 0.001; *F*_SNL(1,28)_ = 7.118, *p* = 0.013) without interaction (*F*_OB × SNL(1,28)_ = 1.61, *p* = 0.215). In the NOB-SNL group, the nuclear GR level was up-regulated compared with that in the NOB-Sham group (*p* = 0.01). The nuclear GR level was dramatically decreased in both OB-SNL group and OB-Sham group compared with their corresponding NOB groups (*p* < 0.001, *p* = 0.009, respectively), showing a reduction of GR nuclear translocation. The purity of cytoplasmic and nuclear fractions was assayed by anti-GAPDH (for cytosolic compartment) or anti-histone H3 (for nuclear compartment) antibodies respectively, as shown in Figure 4I.

Furthermore, Pearson’s correlation analysis was employed to calculate the correlation between spinal nuclear GR expression and the mechanical PWTs or thermal PWLs of rats, respectively. As shown in Figures 4J,K, the spinal nuclear GR expression was negatively correlated with both mechanical PWTs (*r* = −0.5654, *p* < 0.001) and thermal PWLs (*r* = −0.5527, *p* = 0.001). This result revealed that the alteration of nociceptive behaviors of depressed or/and peripheral nerve injury rats was accompanied by changes in GR expression and nuclear translocation in the spinal dorsal horn.

The basal serum corticosterone level in rats were measured and there was a significant effect of SNL (*F*_OB(1,20)_ = 0.602, *p* = 0.447; *F*_SNL(1,20)_ = 7.972, *p* = 0.01) with OB × SNL interaction (*F*_OB × SNL(1,20)_ = 14.12, *p* = 0.001; Figure 4F). The serum corticosterone concentration in OB-Sham group was significantly higher than that in NOB-Sham group (*p* = 0.004) suggesting an increased activity of HPA axis induced by OB, which has been reported in previous studies (Marcilhac et al., 1999; Cryan and Mombereau, 2004). No difference was found in the serum corticosterone concentrations between NOB-SNL group and NOB-Sham group (*p* = 0.516), which is consistent with previous observations (Ulrich-Lai et al., 2006; Alexander et al., 2009; Benedetti et al., 2012). Interestingly, corticosterone level in OB-SNL group was lower than that in OB-Sham group (*p* < 0.001) and NOB-SNL group (*p* = 0.048), indicating a decreased activity of HPA axis, which may due to the exhausted state of HPA axis in OB rats following SNL surgery.

#### The GR Agonist Dexamethasone Eliminated the OB-Mediated Attenuation of Mechanical Allodynia and Thermal Hyperalgesia in Rats with Neuropathic Pain

**Figure 5:**
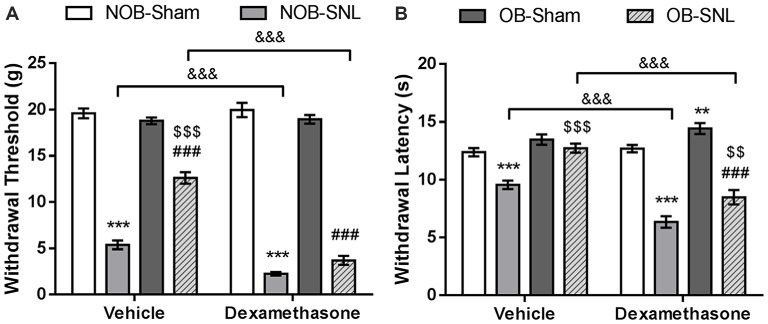
**GR agonist Dex eliminated the OB-induced attenuation of mechanical allodynia and thermal hyperalgesia of neuropathic pain.** Intrathecal administration of Dex (4 μg per rat, daily for 1 week) exacerbated down-regulation of mechanical PWT **(A)** and thermal PWL **(B)** in Dex-NOB-SNL rats compared with those in Veh-NOB-SNL rats and induced significantly lower mechanical PWT and thermal PWL in the Dex-OB-SNL group than those in the Veh-OB-SNL group. The nociceptive behaviors of NOB-Sham rats and OB-Sham rats were not affected by Dex intrathecal administration. **p < 0.01, ***p < 0.001 vs. corresponding NOB-Sham groups; ^###^*p* < 0.001 vs. corresponding OB-Sham groups; ^$$^*p* < 0.01, ^$$$^*p* < 0.001 vs. corresponding NOB-SNL groups; ^&&&^*p* < 0.001 vs. corresponding vehicle groups; *n* = 7 per group.

To demonstrate that spinal GR activity reduction contributes to the attenuation of neuropathic pain caused by OB, GR agonist Dex, proven to promote GR nuclear translocation, was administered intrathecally at 4 μg per rat daily for 1 week which was determined empirically from previous studies (Wang et al., 2004, 2005), starting from the day of SNL surgery. Next, mechanical PWT and thermal PWL were measured. As shown in Figures 5A,B, we found significant effects of Dex on both PWT (*F*_DEX(1,48)_ = 58.488, *p* < 0.001) and PWL (*F*_DEX × SNL(1,48)_ = 73.984, *p* < 0.001 of PWT; *F*_DEX × SNL(1,48)_ = 53.92, *p* < 0.001 of PWL), as well as Dex × OB and Dex × OB × SNL interactions on PWT (*F*_DEX × OB(1,48)_ = 17.316, *p* < 0.001; *F*_DEX × OB × SNL(1,48)_ = 13.464, *p* = 0.001). Compared with the corresponding vehicle group, the mechanical allodynia and thermal hyperalgesia of NOB-SNL rats were aggravated by Dex (*p* < 0.001). Importantly, the attenuation of mechanical allodynia and thermal hyperalgesia in the OB-SNL group was eliminated following the administration of Dex, but not vehicle (*p* < 0.001). While the thermal PWL of the Dex-OB-SNL group was higher than that of the Dex-NOB-SNL group (*p* = 0.001), it still showed an attenuation effect of OB on thermal hyperalgesia. Furthermore, the mechanical PWT and thermal PWL of NOB-Sham rats (*p* = 0.481, *p* = 0.632, respectively) and OB-Sham rats (*p* = 0.059, *p* = 0.132, respectively) were not affected following Dex administration.

These results revealed that spinal GR activation was involved in the development of mechanical allodynia and thermal hyperalgesia of NOB-SNL rats and that decreased GR nuclear translocation, at least in part, contributed to the OB-induced attenuation of neuropathic pain in OB-SNL rats.

### Decreased Spinal BDNF, TrkB and NMDA Receptor Subunit NR2B Expression Levels in Depressed Rats with Neuropathic Pain Were Accompanied by the Attenuation of Nociceptive Hypersensitivity, which Could be Up-Regulated by the GR Agonist Dexamethasone

**Figure 6:**
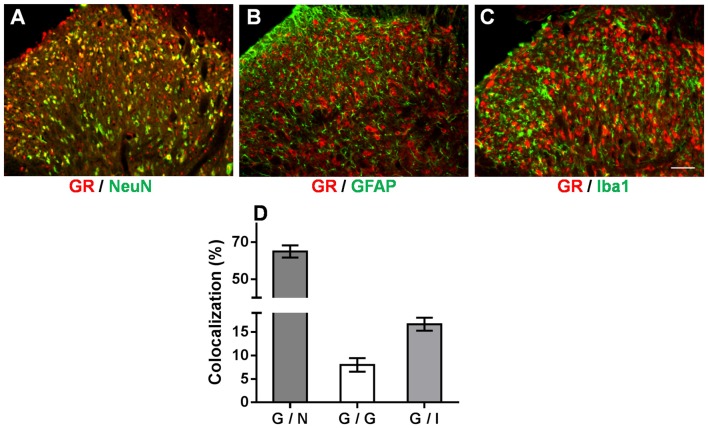
**Immunofluorescence double staining of the GR (red) and cell markers (green) in the spinal dorsal horn of NOB-SNL rats.** GRs were mainly colocalized with NeuN (a marker for neurons; **A**), partly with Iba1 (a marker for microglia; **C**), and rarely with GFAP (a marker for astrocytes; **B**). Colocalization rate of GRs with NeuN, GFAP and Iba1 **(D)**, *n* = 6 sections from three rats per group; scale bar = 50 μm.

We demonstrated that the OB-induced reduction in GR nuclear translocation in the spinal dorsal horn contributed to the attenuation of mechanical allodynia and thermal hyperalgesia in OB-SNL rats. However, how spinal GR participated in this attenuated neuropathic pain behavior in depressive-like rats was still unknown. Accordingly, double immunofluorescence staining was employed to investigate the spinal GR location in NOB-SNL rats, in which GR expression was enhanced and neural glial cells were activated. As Figures 6A–D shows, GR co-expressed with 64.99 ± 3.25% of neurons (Figure 6A), 16.62 ± 1.4% of microglia (Figure 6C), and 7.97 ± 1.45% of astrocytes (Figure 6B). This result revealed that in the spinal dorsal horn GRs were mainly found in neurons, partly in microglia and rarely in astrocytes, suggesting that most of the spinal neurons and some of the glia cells with abnormal GR translocation may be involved in the attenuated neuropathic nociception in depressive-like rats.

**Figure 7:**
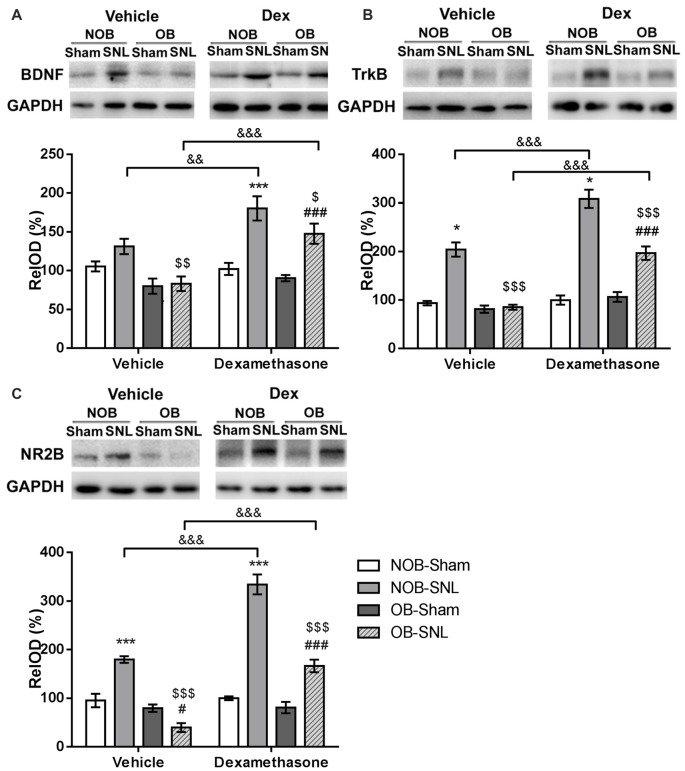
**GR agonist Dex regulated brain-derived neurotrophic factor (BDNF), TrkB and N-methyl-D-aspartate (NMDA) receptor NR2B subunit expression in the spinal dorsal horn of rats with depression and neuropathic pain.** Intrathecal administration of Dex (4 μg per rat, daily for 1 week) exacerbated the enhancement of spinal BDNF **(A)**, TrkB **(B)** and NR2B **(C)** expressions in the Dex-NOB-SNL group compared to the Veh-NOB-SNL group and induced significant up-regulation of these proteins in the Dex-OB-SNL group compared to those in the Veh-OB-SNL group. The protein expression levels of NOB-Sham rats and OB-Sham rats were not affected by Dex. *p < 0.05, ***p < 0.001 vs. corresponding NOB-Sham groups; ^#^*p* < 0.05, ^###^*p* < 0.001 vs. corresponding OB-Sham groups; ^$^*p* < 0.05, ^$$^*p* < 0.01, ^$$$^*p* < 0.001 vs. corresponding NOB-SNL groups; ^&&^*p* < 0.01, ^&&&^*p* < 0.001 vs. corresponding vehicle groups; *n* = 5–6 per group.

It has been established that activation of the BDNF-TrkB pathway in the spinal dorsal horn contribute to the central sensitization of peripheral nerve injury-induced neuropathic pain through microglial-neuronal activation (Beggs and Salter, 2013). In addition, recent studies have shown that CNS GRs can regulate BDNF gene expression in some specific brain areas (Alboni et al., 2011; Gourley et al., 2012; Wosiski-Kuhn et al., 2014). Therefore, the present study investigated whether spinal BDNF and TrkB participated in the OB-induced attenuation of neuropathic pain and whether they were modulated by the GR agonist Dex. Western blotting was employed to analyze BDNF and TrkB protein expression in the spinal dorsal horn from rats that were intrathecally injected with vehicle and Dex as previously described. As shown in Figures 7A,B, OB, SNL and Dex showed significant main effects on BDNF and TrkB expression(BDNF: *F*_OB(1,39)_ = 16.523, *p* < 0.001; *F*_SNL(1,39)_ = 32.067, *p* < 0.001; *F*_DEX(1,39)_ = 17.261, *p* < 0.001. TrkB: *F*_OB(1,39)_ = 50.777, *p* < 0.001; *F*_SNL(1,39)_ = 155.372, *p* < 0.001; *F*_DEX(1,39)_ = 55.596, *p* < 0.001), with OB × SNL and Dex × SNL interactions (BDNF: *F*_DEX × SNL(1,39)_ = 13.421, *p* = 0.001. TrkB: *F*_OB × SNL(1,39)_ = 45.84, *p* < 0.001; *F*_DEX × SNL(1,39)_ = 31.023, *p* < 0.001). In the vehicle groups, there was an increasing trend in BDNF expression (*p* = 0.095) and a significant up-regulation of TrkB expression in NOB-SNL rats (*p* < 0.001) compared with NOB-Sham rats, while the expression of BDNF and TrkB in OB-SNL rats was lower than that in NOB-SNL rats (*p* = 0.002 and *p* < 0.001, respectively) and OB-Sham group (*p* < 0.001). Dex exacerbated this up-regulation of BDNF and TrkB in both the Dex-NOB-SNL group (*p* = 0.001, *p* < 0.001, respectively) and the Dex-OB-SNL group (*p* < 0.001) compared with their corresponding vehicle groups. Interestingly, the expression of BDNF and TrkB in the Dex-OB-SNL group was still lower than that in the Dex-NOB-SNL group (*p* = 0.028, *p* < 0.001, respectively). In addition, Dex did not affect spinal BDNF or TrkB expression in NOB-Sham rats (*p* = 0.828, *p* = 0.772, respectively) or OB-Sham rats (*p* = 0.471, *p* = 0.133, respectively).

The NR2B subunit of the *N*-methyl-D-aspartate (NMDA) receptor in the neurons of spinal superficial laminae participates in the nociceptive transmission and contributes to the maintenance of neuropathic pain. It has been reported that the activation of the NR2B subunit was modulated by BDNF-TrkB signaling in the spinal dorsal horn following peripheral nerve injury (Geng et al., 2010). Hence, we analyzed NR2B subunit expression, as well. As shown in Figure 7C, OB, SNL and Dex showed significant main effects on NR2B expression (*F*_OB(1,39)_ = 106.117, *p* < 0.001; *F*_SNL(1,39)_ = 119.746, *p* < 0.001; *F*_DEX(1,39)_ = 74.416, *p* < 0.001), with OB × SNL and Dex × SNL interactions (*F*_OB × SNL(1,39)_ = 67.046, *p* < 0.001; *F*_DEX × SNL(1,39)_ = 68.312, *p* < 0.001). In the vehicle groups, NR2B expression in NOB-SNL rats was higher than that in the NOB-Sham group (*p* < 0.001), while NR2B expression in OB-SNL rats was lower than that in the NOB-SNL group (*p* < 0.001) and the OB-Sham group (*p* = 0.021). Compared with vehicle treatment, Dex treatment aggravated the up-regulation of NR2B induced by SNL in both Dex-NOB-SNL and Dex-OB-SNL groups (*p* < 0.001), but expression in the Dex-OB-SNL group was still lower than that in the Dex-NOB-SNL group (*p* < 0.001). Moreover, Dex treatment did not influence the spinal NR2B expression in NOB-Sham rats (*p* = 0.793) or OB-Sham rats (*p* = 0.929).

These findings showed that decreased spinal BDNF, TrkB and NR2B expression was involved in the OB-induced attenuation of neuropathic pain. The GR agonist Dex enhanced nociceptive sensitivity and BDNF, TrkB and NR2B expression in the spinal dorsal horn of both NOB-SNL rats and OB-SNL rats but not Sham rats.

## Discussion

In the present study, we found that OB-induced depressive-like behavior and SNL-induced neuropathic pain behavior were associated with alterations in GR expression and nuclear translocation in the spinal dorsal horn. The mechanical allodynia and thermal hyperalgesia of neuropathic pain were attenuated by OB, with decreased serum corticosterone level and reduced GR expression and nuclear translocation, as well as reduced BDNF, TrkB, NR2B subunit expression in the spinal dorsal horn. The GR agonist Dex eliminated the attenuating effect of depression on nociceptive hypersensitivity in OB-SNL rats and aggravated neuropathic pain in NOB-SNL rats, with enhanced BDNF, TrkB and NR2B subunit expression in the spinal dorsal horn. Interestingly, Dex showed no effect on nociceptive behaviors or the expression of the aforementioned proteins in NOB-Sham rats and OB-Sham rats.

### Olfactory Bulbectomy Induces Depressive-Like Behavior and Attenuates Nociceptive Response in Rats with Neuropathic Pain

Among various animal depression models, OB is usually performed on rodents, mostly on rats, to induce a series of behavioral, neurochemical and neuroendocrine alterations related to those changes in depression patients (van Riezen and Leonard, 1990; Kelly et al., 1997). Sometimes OB is questioned for the obscure mechanism of its use as a depression model, but it is thought to be one of the best reliable models for predicating the efficacy of chronic antidepressants treatment (Willner and Mitchell, 2002; Cryan and Mombereau, 2004). In the present study, we observed that OB induced hyperactivity in open field test which is related to hyperactive responses to stress and increases in defensive behavior (Stock et al., 2001), decreased sucrose consumption which suggests anhedonia symptoms (Romeas et al., 2009), and decreased bodyweight gain which indicates loss of appetite. Although the mechanism of OB induced abnormal behaviors is still not fully understood, these symptoms were not simply a consequence of loss of smell because peripheral anosmia (e.g., zinc sulfate-induced anosmia) failed to produce the same effects (Sieck and Baumbach, 1974; Mar et al., 2000). These alterations are believed to be due to complex neuronal reorganization in various subcortical limbic regions including amygdala and hippocampus (Kelly et al., 1997).

The influence of depression on pain perception is controversial. However, there are an increasing number of clinical and laboratory studies indicating that the sensitivity of the response to exogenous nociceptive stimuli is reduced in patients with depression or a depressive-like state. Major depression patients showed decreased sensitivity to noxious stimuli applied to their skin (Lautenbacher et al., 1994; Bär et al., 2006). Our previous study showed that depressive-like state induced by unpredictable chronic mild stress (UCMS) resulted in hypoalgesia to thermal stimuli in rats with completed Freund’s adjuvant (CFA)-induced chronic inflammatory pain (Shi et al., 2010b), as well as increased mechanical PWT and thermal PWL in rats with neuropathic pain (Shi et al., 2010a). In the present study we found that following SNL surgery OB-induced depressive-like rats showed less mechanical allodynia and thermal hyperalgesia than non-depressed rats, which indicated that depressive-like state may attenuate the sensitivity to nociceptive stimuli in rats with neuropathic pain. These results are consistent with previous findings. However, Bravo et al. (2014) found that there was no difference in mechanical allodynia between rats experiencing CMS combined with sciatic nerve chronic constriction injury (CCI) and non-CMS rats with CCI. Burke et al. (2013, 2014) reported normal mechanical allodynia and thermal hyperalgesia in OB-SNL rats. These conflicting results may be related to differences among neuropathic pain models, experimental paradigms or animal handling methods before the behavior tests.

### The Role of Spinal GR Nuclear Translocation in Attenuating Effect of Depression on Neuropathic Pain

GR is a classical nuclear receptor. When binding with glucocorticoids, GRs translocate from cytoplasm into nucleus to mediate transcriptional activation/repression of target genes, participating in the initiation and development of various physiological and pathological processes, including major depression and neuropathic pain (Mao, 2005; Kadmiel and Cidlowski, 2013; Madalena and Lerch, 2016). Many studies on major depression patients or depressive-like animal models have reported abnormal GR expression and function in the pituitary or hippocampus, amygdala, prefrontal cortex and other brain areas, which was considered one of the important reasons for HPA axis hyperactivity and glucocorticoid resistance (Revollo and Cidlowski, 2009; Anacker et al., 2011a). Some studies have found that GR nuclear translocation in hippocampus and PFC was impaired in CMS-induced depressive-like rats, which could be reversed by chronic treatment with antidepressants (Guidotti et al., 2013; Xing et al., 2015). Studies *in vitro* reported that the tricyclic antidepressant desipramine facilitated GR nuclear translocation in mouse fibroblasts (Pariante et al., 1997), and SSRI antidepressant sertraline increased GR transactivation in human hippocampal progenitor cells (Anacker et al., 2011b). These findings suggests that impaired GR translocation in brain is associated with depressive-like state and GR is a potential target for antidepressants treatment. However, alterations of GR translocation in the spinal dorsal horn in depressive-like rodents remain poorly understood. In the present study, we found significant a decrease in GR nuclear translocation in the spinal dorsal horn of OB-Sham and OB-SNL rats, and an obvious increases in GR expression and translocation in the spinal dorsal horn of NOB-SNL rats. The positive correlation between spinal nuclear GR level and nociceptive behaviors of OB/NOB-SNL/Sham rats suggests that spinal GRs may be involved in the development of hypersensitivity in neuropathic pain nociception. We speculate that OB-induced decreased spinal GR nuclear translocation may, at least in part, account for the attenuation of mechanical allodynia and thermal hyperalgesia in OB-SNL rats.

Therefore, GR agonist Dex was used to confirm the role of spinal GRs in the process by which depression attenuates neuropathic pain. We observed that Dex administered intrathecally aggravated mechanical allodynia and thermal hyperalgesia in NOB-SNL rats and eliminated the attenuating effect of depression on neuropathic pain in OB-SNL rats. In a previous study, spinal GR protein expression was increased in spinal nerve injury (SNI) rats, and this expression could be exacerbated by the synthetic glucocorticoid betamethasone (Wang Q. S. et al., 2013). Furthermore, Wang et al. (2004) found a significant elevation of spinal GR mRNA and protein expression was associated with neuropathic pain behaviors following CCI surgery. GR agonist Dex (i.t.) exacerbated the development of thermal hyperalgesia and mechanical allodynia in CCI rats, while the GR antagonist RU486 or GR antisense oligonucleotide (i.t.) abolished it. Our results were consistent with these findings and demonstrated that GR activation is essential for the development of mechanical allodynia and thermal hyperalgesia induced by peripheral nerve injury. We hypothesized that decreased spinal GR nuclear translocation caused by OB may interrupt GR activation, which is necessary for nociceptive hypersensitivity following peripheral nerve injury, and contributes to the attenuating effect of depression on allodynia and hyperalgesia of neuropathic pain.

### The Expression of Spinal BDNF, TrkB and NR2B May be Modulated by Decreased GR Activity and Involved in the Hypoalgesic Effect of Depressive-Like State on Neuropathic Pain

It is well known that spinal BDNF-TrkB signaling contributes to the initiation and development phase of neuropathic pain (Khan and Smith, 2015). Following SNI, increased expressions of BDNF and its receptor TrkB in the spinal dorsal horn are highly correlated with the onset of neuropathic pain behavior (Kerr et al., 1999; Geng et al., 2010). The activation of NR2B subunit of NMDA receptor mediated by BDNF is related with the maintenance phase of neuropathic pain. The selective NR2B antagonist Ro25-6981 significantly attenuated mechanical allodynia and thermal hyperalgesia induced by peripheral nerve injury (Geng et al., 2010). In the present study, SNL-induced up-regulation of BDNF, TrkB and NR2B expressions were eliminated by the depressive-like state induced by OB. The intrathecal administration of Dex aggravated mechanical allodynia and thermal hyperalgesia in depressed and non-depressed SNL rats and increased the spinal expression of BDNF, TrkB and NR2B. These results suggest that the down-regulation of BDNF, TrkB and NR2B associated with depression-induced reduction of GR nuclear translocation may contribute to the attenuation of neuropathic pain behavior in depressed SNL rats and that spinal BDNF-TrkB signaling is possibly modulated by GR activation following intrathecal injection of Dex in rats with neuropathic pain.

Several studies have found that chronic stress-induced depressive-like behavior was associated with a reduction in GR and BDNF expression in the hippocampus, which is responsible for the alterations in hippocampal neural plasticity (Alboni et al., 2011; Xing et al., 2015). Antidepressants could protect hippocampal neurons and reverse these alterations (Anacker et al., 2011b; Tanti and Belzung, 2013). It has been demonstrated that increased GR activity leading to activation of the NR1/NR2 subunits of NMDA receptor in spinal dorsal horn contributed to neuropathic pain in rats with peripheral nerve injury (Wang et al., 2005; Alexander et al., 2009; Le Coz et al., 2014; Zhang et al., 2014). However, there is little evidence on the relationship between spinal GR and BDNF signaling under conditions of depression combined with neuropathic pain. Our findings provide a new perspective for understanding the etiological and pathological mechanisms of how depression affects neuropathic pain.

### The Differential Effect of GR Agonist Dexamethasone on the Nociception of SNL Rats and Sham Rats

Interestingly, we observed that intrathecal treatment with Dex aggravated the mechanical allodynia and thermal hyperalgesia of SNL rats but did not alter the nociceptive behavior of Sham rats in both OB and NOB groups. The expressions of spinal BDNF, TrkB and NR2B were also modulated by Dex the same way as above. This differential effect of GR agonist or antagonist on pathological pain in rodents and their corresponding control group was also reported in other studies (Wang et al., 2004; Maiaru et al., 2016). Because of the evidence that GRs can exert anti-inflammatory or pro-inflammatory effects under different situations (Cruz-Topete and Cidlowski, 2015), we speculate that spinal GRs may participate in the regulation of pro-nociceptive pathway following external noxious stimulation such as nerve injury or inflammation, whereas a completely different signal pathway under normal circumstances. Therefore, it is possible that Dex promotes the pro-nociceptive pathway through GR activation in NOB-SNL and OB-SNL rats, leading to aggravation of nociceptive hypersensitivity and spinal BDNF-TrkB signaling enhancement. On the other hand, the spinal GR activation in NOB-Sham and OB-Sham rats may enhance its related signaling pathways, which may be independent of pro-nociceptive signaling, resulting in unaltered nociception and BDNF-TrkB signaling following Dex treatment.

### The Potential Role of Supraspinal Pain Modulation in the Attenuating Effect of Depression on Neuropathic Nociception

The endogenous descending inhibitory system of pain in brainstem projects axons to the spinal dorsal horn to mediate pain sensation, and receives the direct neural projection or indirect modulation from several pain related brain regions including hypothalamus, hippocampus, amygdala and frontal cortex in the process of pain perception (Heinricher et al., 2009; Kwon et al., 2014). Previous studies have found disorders of serotonergic, noradrenergic or glutamatergic transmission in the mid-brain and forebrain of OB-induced depressed rats (Redmond et al., 1997; Song and Leonard, 2005), and intraperitoneal treatment with 5-HT1A receptor antagonist eliminated hypoalgesia caused by depressive-like state (Wang et al., 2016). These findings suggest the possibility that the serotonergic descending inhibitory pathway may be related to the nociceptive observed in depression. In this study we discovered the restoration effect of Dex on the allodynia and hyperalgesia of depressed SNL rats, but the thermal hypersensitivity and spinal expression of BDNF, TrkB and NR2B were still lower in the Dex-OB-SNL rats than those in the Dex-NOB-SNL rats. It is possible that when activated spinal GR signaling contributes to exacerbated nociceptive transmission in the spinal dorsal horn of Dex-OB-SNL rats, whereas enhanced serotonergic signaling through the brainstem-spinal descending pain pathway may have an opposite function, resulting in lower nociceptive hypersensitivity and related protein expression than those in Dex-NOB-SNL rats.

### The Potential Gender Diversity Under Stress and Neuropathic Pain State

It is documented that female rats revealed enhanced CORT secretion in basal and stress state (Kitay, 1961; Critchlow et al., 1963). What’s more, a recent study found that mechanical allodynia of neuropathic pain was mediated by different spinal immune cells in female and male mice: adaptive immune cells (likely T lymphocytes), but not microglia, were required in the development of nociceptive hypersensitivity in female mice with neuropathic pain (Sorge et al., 2015). In our present study we found that spinal GR not only expressed in neurons but also in microglia of male rats (Figure 5) which suggests microglia may participated in the depression induced hypoalgesia. So the influence and neural mechanism of depressive like state on neuropathic pain in female rats may be quite different from male rats, which need to be carefully explored and discussed in future.

### The Potential Epigenetic and Translocational Modulators of GR Activity

Although our results showed that the changes in the BDNF signaling pathway mediated by decreased GR activity in the spinal dorsal horn may contribute to the attenuation of the mechanical allodynia and thermal hyperalgesia in the depressed peripheral nerve injury rats, the mechanism of its upstream regulation on decreased spinal GR expression and nuclear translocation is still unclear. The transcription and expression of NR3C1 gene, which encoded GR protein, are primarily modulated by GR negative feedback (Oakley and Cidlowski, 2013). At present, studies on the epigenetics of major depression have shown that hypermethylation of the hippocampal NR3C1 exon1 promoter decreases GR protein expression and causes HPA axis dysfunction (Farrell and O’Keane, 2016). However, it is not clear whether this hypermethylation is related to chronic pain. Alternative splicing of exon 9 of the NR3C1 gene primary transcript generates two GR isoforms: GRα, which mediates classical glucocorticoid-induced transcriptional activity, and GRβ, which does not exhibit transcriptional activity and inhibits GRα (Oakley and Cidlowski, 2013). Although GRα is much more abundant than GRβ in almost all tissues and cell types, it has been shown that GRα/GRβ ratio is decreased in monocytes or the frontal cortex and amygdala of major depression patients (Pandey et al., 2013; Grosse et al., 2015). Moreover, Maiaru et al. (2016) reported that spinal GRβ mRNA was increased in rats with CFA-induced chronic inflammatory pain. Hence, the alteration of GRα/GRβ ratio in spinal dorsal horn may be relevant to the mechanism of depression affecting chronic pain processes, which provides an interesting direction to explore in future research.

GR nuclear translocation is mediated by many factors, including the chaperone complex that binds the GR in the cytoplasm (Sasuga et al., 1997; Binder, 2009), phosphorylation of GR protein serine residues by various kinases (Treviño and Weigel, 2013), and cytokines that interact with GR signaling (Pace and Miller, 2009; Brkic et al., 2016). Among them, FK506 binding protein (FKBP5, also called FKBP51), which is one component of the chaperone complex binding with GR, maintains the transcriptionally inactive conformation of GRs to repress their nuclear translocation and downstream gene expression (Binder, 2009). Several studies have demonstrated that FKBP5 is associated with major depression and chronic pain. Furthermore, some clinical studies have suggested that polymorphisms of FKBP5 gene could be used as a biological indicator for the vulnerability and antidepressants responsiveness to major depression (Hartmann et al., 2012; Menke et al., 2012). Lukic et al. (2015) found that the accumulation of cytoplasmic GRs was related to the elevation of FKBP5 in the lymphocytes of depressed patients. Guidotti et al. (2013) have observed that increased FKBP5 expression was associated with decreased GR translocation in the hippocampus of CMS-induced depressive-like rats, which was confirmed by Xing et al. (2015). Maiaru et al. (2016) discovered that intrathecal administration of the specific FKBP5 inhibitor SAFit2, global deletion FKBP5 by gene knockout, or local silencing with FKBP5 siRNA significantly attenuated the mechanical allodynia associated with CFA-induced chronic inflammatory pain via GR signaling. These findings indicated that spinal FKBP5 may be one of the important factors mediating GR signaling in the process by which depression attenuates the allodynia and hyperalgesia of neuropathic pain. Further study of these specific mechanisms is worth considering in the future.

## Conclusion

We found that decreased GR expression and translocation, which mediated BDNF, TrkB and NR2B down-regulation in the spinal dorsal horn, contributed to the attenuating effect of OB-induced depression on mechanical allodynia and thermal hyperalgesia of neuropathic pain. The GR agonist Dex eliminated this attenuating effect and enhanced BDNF-TrkB signaling, providing a novel perspective for understanding the hypoalgesic effect of depressive-like state on neuropathic pain in rats.

## Author Contributions

This study was designed by XW and FL; experiments in this study were performed by XW and YS; data was analyzed by XW and YS; this article was written by XW; the design and experiments of this study were supervised by FL; XW and FL revised the manuscript.

## Conflict of Interest Statement

The authors declare that the research was conducted in the absence of any commercial or financial relationships that could be construed as a potential conflict of interest.
